# Traumatic transmesenteric hernia leading to ischemic enterocolitis in an adolescent - case report

**DOI:** 10.1016/j.ijscr.2025.111314

**Published:** 2025-04-18

**Authors:** Zahin Shahriar, Deluwar Hussen, Saidul Mustofa

**Affiliations:** a Dhaka Medical College, Bangladesh; b M Abdur Rahim medical college, Bangladesh

**Keywords:** Traumatic transmesenteric hernia, Ischemic enterocolitis, Acute intestinal obstruction, Internal hernia, Pediatric emergency, Exploratory laparotomy

## Abstract

**Introduction and importance:**

Traumatic transmesenteric hernia (TTMH) is an exceptionally rare condition with high morbidity and mortality, particularly in children. It can cause life-threatening complications such as ischemic enterocolitis. Internal hernias, including transmesenteric types, are difficult to diagnose preoperatively and are often discovered during emergency laparotomy.

**Case presentation:**

A 13-year-old girl presented with acute abdominal pain, bilious vomiting, and hypovolemic shock. She had sustained blunt abdominal trauma from a fall two weeks earlier. Initial resuscitation and imaging raised concerns for acute intestinal obstruction. Due to clinical deterioration, an emergency exploratory laparotomy was performed, revealing a transmesenteric hernia with 120 cm of ischemic ileum. Bowel resection and a double-barrel ileostomy were performed. Histopathology confirmed ischemic enterocolitis secondary to the hernia. The patient recovered well and later underwent successful ileostomy reversal.

**Discussion:**

Transmesenteric hernias are rare, typically congenital or post-surgical, with traumatic cases being extremely uncommon. Diagnosis is challenging due to a lack of specific clinical findings. Delayed intervention increases the risk of bowel ischemia and mortality.

**Conclusion:**

This case highlights the diagnostic difficulty of TTMH and emphasizes the need for a high index of suspicion in trauma patients presenting with acute abdomen. Educating patients with trauma about warning signs & follow up is the recommendation.

## Highlights

• Preoperative diagnosis of transmesenteric hernia is challenging due to the lack of specific clinical or radiological markers.
• Early surgical intervention is crucial to prevent bowel ischemia, necrosis, and fatal complications.
• This case emphasizes the importance of maintaining a high index of suspicion in trauma patients presenting with acute abdomen**.**

## 1 Introduction

Internal hernia is described as a condition whereby a viscera herniates through a defect in the mesentery or in the peritoneum through other means [1].It is classified in terms of location, including para-duodenal, peri-cecal, foramen of Winslow, transmesenteric, trans-mesocolic, inter-sigmoid, and retro-anastomotic [2].

An exceedingly infrequent occurrence, internal hernias are said to occur in 0.2–0.9 % of the general population [3]. Most of these result from adult post-surgical complications, post-laparotomy, peritoneal infection, or post-traumatic events. Among children, the major cause of internal hernia is when intestinal contents protrude via congenital mesenteric defect. It occurs in 0.6–5.8 % of all cases with small bowel obstruction among children [4]. Transmesenteric hernia is an intra-abdominal hernia that does not have a sac and represents about 5–10 % of all childhood internal hernias [5]. Trans-mesenteric as it can be was first recorded by Carl Maximilian Rokitansky in the year 1836 [6]. The majority of mesenteric defects leading to herniation are located in the ileocecal mesentery, and they measure between 2 and 5 cm wide and trap a loop of ileum.

We report a case of a transmesenteric hernia that presented as abdominal pain and vomiting in an adolescent with a significant time lapse between the traumatic event and the onset of severe symptoms from abdominal trauma caused by a fall in height, with a final diagnosis of ischemic enterocolitis. Transmesenteric hernias are already very rare, but it serves as a unique instance where trauma is the cause.

## 2 Methods

This case report has been prepared following the Surgical Case Report (SCARE) 2023 guidelines to ensure transparency and comprehensive reporting [7].

## 3 Case presentation

A 13-year-old girl with no significant medical history presented to the emergency department with severe, colicky abdominal pain, bilious vomiting, and abdominal distension that had persisted for one day. She had not passed stool or flatus for 24 h and reported cessation of urination over the last 12 h. Approximately 15 days prior, she experienced a fall onto her abdomen, causing mild, diffuse pain that resolved within four days with analgesics, without the need for hospital admission.

Upon examination, the patient was restless and in hypovolemic shock. Her pulse was initially not palpable, and her blood pressure was unrecordable until stabilized to 80/60 mmHg after resuscitation. Respiratory rate was 28 breaths/min with rapid, shallow breathing. Her abdomen was distended, tender, and rigid, with absent bowel sounds. A digital rectal examination was unremarkable, and other systemic examinations revealed no abnormalities.

### 3.1 Clinical approach and differential diagnosis

The initial differential diagnosis included acute intestinal obstruction with hypovolemic shock, acute pancreatitis, and perforation of a gas-containing hollow viscus. Recognizing the severity of the case, an immediate resuscitative protocol was initiated, including fluid replacement via two wide-bore IV cannulas, nasogastric decompression, bi-channel Foley catheter placement for urine output monitoring, and administration of IV antibiotics, anti-ulcerants, analgesics, and anti-emetics.

The patient's condition showed slight improvement with resuscitation, allowing for further investigations (Table 1). However, her condition worsened later, necessitating ICU transfer and intubation due to hypoxemia and shock unresponsive to fluids alone.

**Table 1:** Laboratory investigations.

Investigation	Result	Reference range
Hemoglobin (Hb)	10.5 g/dL	12–16 g/dL (female)
White Blood Cell Count	22,650 /cumm	4–11 × 10^9/L
Neutrophil	86 %	52–62 %
Lymphocyte	10 %	40–50 %
Platelet Count	400,000 /cumm	150–400 × 10^9/L
Erythrocyte Sedimentation Rate (ESR)	20 mm/h	0–20 mm/h
Sodium (Na)	133 mmol/L	136–148 mmol/L
Potassium (K)	3.0 mmol/L	3.5–5.2 mmol/L
Chloride (Cl)	93 mmol/L	96–108 mmol/L
Serum Amylase	160 U/L	30–110 U/L
Serum Creatinine	1.17 mg/dL	0.4–1.2 mg/dL (female)

### 3.2 Radiological findings

**Fig. 1:**
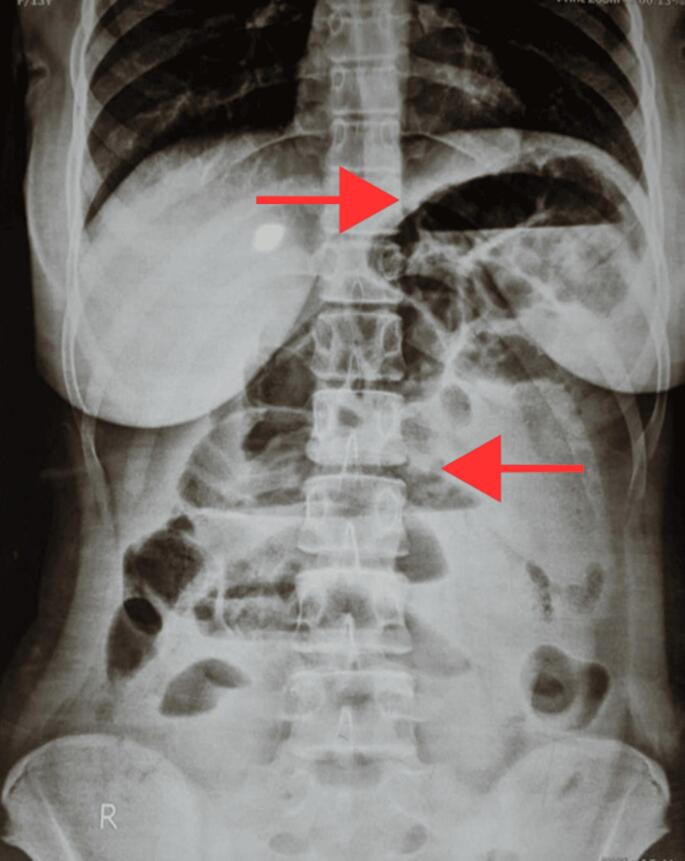
Abdominal X-ray showing central multiple air-fluid levels.

**Fig. 2:**
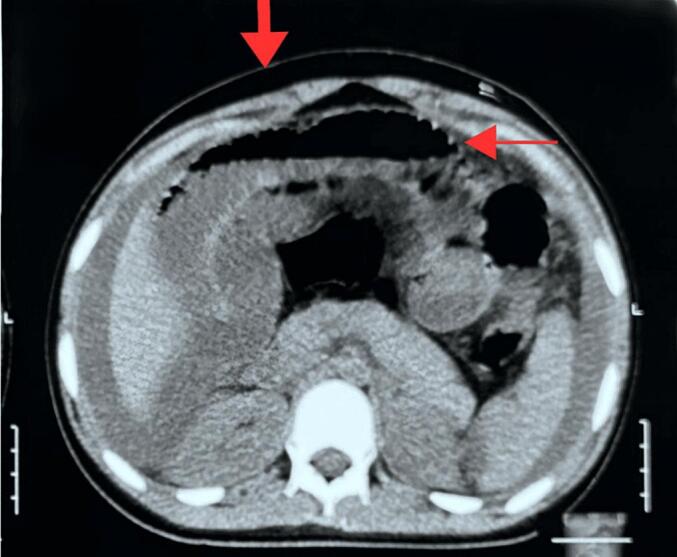
Single slide from contrast-enhanced CT scan, with marked lesions showing ascites and bowel distension.

Plain abdominal X-ray in erect posture (Fig. 1) showed multiple centrally-placed air-fluid levels in a step-ladder pattern, suggestive of small bowel obstruction, without free air under the diaphragm. Given her worsening condition, a contrast-enhanced CT scan was performed (Fig. 2), revealing distended bowel loops, ascites, and no solid organ injury. However, the ischemic changes were not evident at that time.

### 3.3 Surgical intervention

**Fig. 3:**
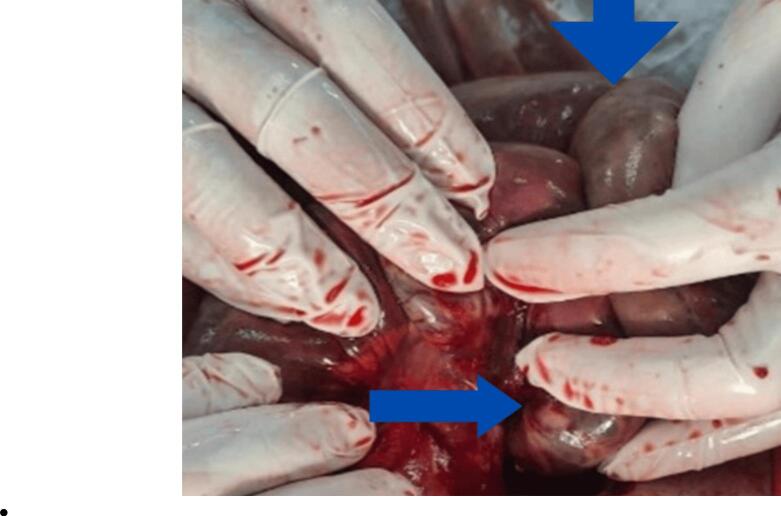
Per-operative finding of gangrenous bowel.

**Fig. 4:**
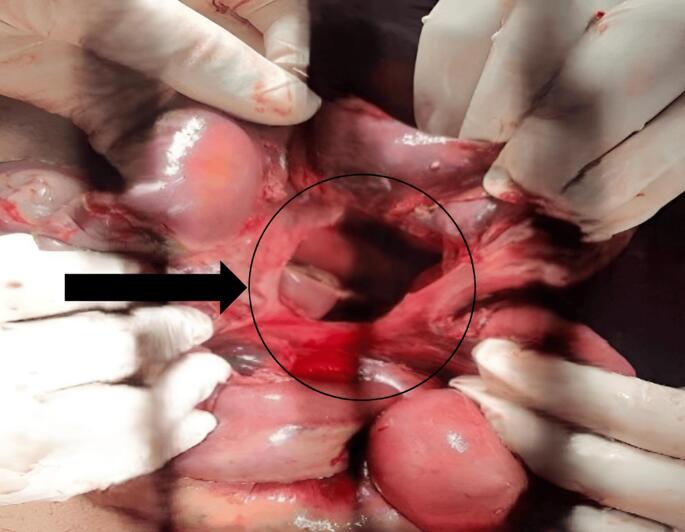
Mesenteric defect after resection of gangrenous bowel.

After counseling the patient's family about the high-risk nature of the surgery, an exploratory laparotomy was performed. Intraoperatively, we found approximately 2 l of hemorrhagic peritoneal fluid and a transmesenteric hernia through a defect at the root of the mesentery. The hernia entrapped around 120 cm of ischemic ileum, 250 cm distal to the duodenojejunal junction and 150 cm proximal to the ileocecal valve. The gangrenous segment was resected, and a double-barrel ileostomy was performed (Fig. 3, Fig. 4). The mesenteric defect was closed, and a pelvic drain was placed. The diagnosis of trans mesenteric hernia was made intraoperatively with the aetiology of trauma. The management decision was based on the delayed presentation of the case and the patient intraoperative findings.

Postoperatively, the patient was managed in the ICU and gradually weaned off ventilator support. Histopathological examination of the resected ileum confirmed ischemic necrosis without malignancy. The patient recovered well, with plans for ileostomy closure after stabilization. After six weeks ileostomy was reversed and now, she is completely healthy.

## 4 Discussion

Internal hernias are considered an uncommon cause of acute intestinal obstruction. There were no evident clinical signs indicating the existence of internal abdominal hernia, but the main complaint might be abdominal pain and distension, which is non-specific. As a result, the majority of them are diagnosed intraoperatively.

Congenital abnormalities in the mesentery can be linked to conditions such as bowel obstruction, intestinal twisting, duplicated bowels, Hirschsprung's disease, and cystic fibrosis, indicating a cause. This defect typically occurs during the stages of development due to factors like changes in the mesentery structure, inadequate blood supply in certain areas, rapid growth of a section of the mesentery, or pressure from the colon as it moves into the yolk sac. Similar to any hernia, this condition can result in strangulation or twisting, leading to tissue death. While internal hernias are causes of obstruction accounting for only 0.5–3 % of cases, complications like twisting or strangulation from transmesenteric herniation are more common, with rates ranging from 30 to 40 % [8,9].It is crucial for medical professionals to promptly diagnose and treat this condition, as mortality rates can be as high as 45 % if treated late or 100 % if left untreated altogether [9]. Therefore, emergency physicians should be vigilant about considering this possibility when evaluating patients with symptoms like pain and vomiting.

Obstructive symptoms and spontaneous resolution could possibly be the presentation of Treves' field transmesenteric hernia in patients without predisposing factors of intraabdominal adhesion [10].

Furthermore, because transmesenteric hernia is an uncommon cause of intestinal obstruction and strangulation in adults, detecting it prior to surgery is extremely difficult. Aside from the possibility of a clinical diagnosis, the lack of specific radiographic or laboratory evidence effectively renders all IAHs undiagnosable preoperatively; misdiagnosis with a delay in investigation may result in intestinal ischemia and mortality. The majority of these cases are discovered quite late, usually at the time of laparotomy, after a certain clinical presentation and/or further investigations that show intestinal obstruction. This causes life-threatening bowel ischemia. Because of this diagnostic quandary, a portion of the small intestine was also deemed non-viable in our case.

In low resource setting patients refuses to do costly imaging tests like CT scan for screening when patient is apparently stable. A time-consuming diagnostic workup before surgery may be dangerous for these individuals because intestinal obstruction progresses quickly to bowel ischemia. Imaging examinations and a comprehensive clinical evaluation are required. CT scan is the gold standard diagnostic for internal hernia although detecting a transmesenteric hernia might be problematic. A CT scan might increase suspicion of transmesenteric hernia, despite its lack of specificity. The characteristic signs of transmesenteric hernia is described as small intestinal dilatation, a cluster of small bowel loops, colon displacement in the middle, a lack of omental fat covering the cluster, and displacement of the mesenteric trunk [1].

We conducted a literature search using the MEDLINE database and discovered only two cases of transmesenteric post-traumatic hernia [11,12]. Despite the efforts of surgeons to close the defects that have been created, they may be incompletely closed or may experience a collapse or a dragging of the suture material through the meso-colic fat [11]. In both of them, bowel resection & repair of defect was done only. But in our case we did a double-barrel ileostomy after resection & repair of defect. This is the better approach as we are not sure of viable length of bowel due to ischemia and let the distal anastomosis heal. One stoma is for drainage of stool & other for mucus. Septic patients have been found to have better outcome & less complications in this approach [13].

Our patient had never had surgery before and had no history of abdominal pain that resolved on its own. Rather, she presented to us with a delayed manifestation of abdominal trauma caused by a fall from a height. Furthermore, the CT abdomen revealed no significant findings other than dilated bowel loops with ascites. Emergency surgical intervention is required to avoid high mortality from intestinal strangulation and necrosis. However, despite recent advances in diagnostic technologies, it's not feasible in low resource settings. Early laparotomy with reduction and closure of the defect is the mainstay of management. If the bowel is found to be tightly trapped, the defect should be expanded, and necrosed portion resected. The entire bowel should be searched for the presence of any other potential defects and should be closed also to prevent future occurrences.

## 5 Conclusion

Given that early recognition of internal hernia symptoms can be life-saving, educating patients on warning signs could be an essential preventive measure. Imaging like CT scans should be considered early on to rule out intestinal obstruction in patients with abdominal trauma when deemed feasible.

## Statement of consent

Written informed consent was obtained from the patient's parents/legal guardian for publication and any accompanying images. A copy of the written consent is available for review by the Editor-in-Chief of this journal on request.

## Ethical approval

Approved.

## Funding

This work is not funded.

## Author contribution

All authors attest that they meet the current ICMJE criteria for Authorship. Role of authors according to CRediT taxonomy are mentioned below:

Author 1: Dr. Zahin Shahriar Role: Conceptualization, Data curation, Project administration, Resources, Supervision, Writing - original draft.

Author 2: Dr. Deluwar Hussen Role: Methodology, Software, Validation, Writing - original draft.

Author 3: Dr. Saidul Mustofa Role: Project administration, Resources, Supervision, Validation, Visualization and was directly involved in patient management (OT assist & follow-up).

## Guarantor

Data will be available on reasonable request from the corresponding author.

## Research registration number

Not applicable for this case report.

## Declaration of Generative AI and AI-assisted technologies in the writing process

For improving readability Grammarly and for proofreading ChatGPT has been used.

## Conflict of interest statement

None declared.

## Data Availability

Data will be available on reasonable request from the corresponding author.
